# Silencing of beta-carotene hydroxylase increases total carotenoid and beta-carotene levels in potato tubers

**DOI:** 10.1186/1471-2229-7-11

**Published:** 2007-03-02

**Authors:** Gianfranco Diretto, Ralf Welsch, Raffaela Tavazza, Fabienne Mourgues, Daniele Pizzichini, Peter Beyer, Giovanni Giuliano

**Affiliations:** 1 ENEA, Casaccia Research Center, PO Box 2400, Roma 00100AD, Italy; 2 Faculty for Biology, Universität Freiburg, Schänzlestrasse 1, 79104 Freiburg, Germany

## Abstract

**Background:**

Beta-carotene is the main dietary precursor of vitamin A. Potato tubers contain low levels of carotenoids, composed mainly of the xanthophylls lutein (in the beta-epsilon branch) and violaxanthin (in the beta-beta branch). None of these carotenoids have provitamin A activity. We have previously shown that tuber-specific silencing of the first step in the epsilon-beta branch, *LCY-e*, redirects metabolic flux towards beta-beta carotenoids, increases total carotenoids up to 2.5-fold and beta-carotene up to 14-fold.

**Results:**

In this work, we silenced the non-heme beta-carotene hydroxylases *CHY1 *and *CHY2 *in the tuber. Real Time RT-PCR measurements confirmed the tuber-specific silencing of both genes *. CHY *silenced tubers showed more dramatic changes in carotenoid content than *LCY-e *silenced tubers, with beta-carotene increasing up to 38-fold and total carotenoids up to 4.5-fold. These changes were accompanied by a decrease in the immediate product of beta-carotene hydroxylation, zeaxanthin, but not of the downstream xanthophylls, viola- and neoxanthin. Changes in endogenous gene expression were extensive and partially overlapping with those of *LCY-e *silenced tubers: *CrtISO*, *LCY-b *and *ZEP *were induced in both cases, indicating that they may respond to the balance between individual carotenoid species.

**Conclusion:**

Together with epsilon-cyclization of lycopene, beta-carotene hydroxylation is another regulatory step in potato tuber carotenogenesis. The data are consistent with a prevalent role of *CHY2*, which is highly expressed in tubers, in the control of this step. Combination of different engineering strategies holds good promise for the manipulation of tuber carotenoid content.

## Background

The biofortification of potato is a viable strategy for the eradication of a series of nutritional deficiencies, since this crop stands fourth, among staple foods, in yearly *per capita *consumption. Several efforts are under way for the metabolic engineering of potato carotenoid content [1-3]. In a companion paper, we reported the results of the tuber-specific silencing of the first dedicated step in lutein biosynthesis, *LCY-e *[3]. This resulted in increases of β-carotene (up to 14-fold) and of total carotenoids (up to 2.5-fold). No changes in carotenoid content, or in endogenous carotenoid gene expression, were observed in leaves, indicating that, in agreement with previous reports [1], gene silencing remains confined in tubers.

Encouraged by this result, we decided to silence a second important regulatory step in carotenogenesis, the hydroxylation of β-carotene. This step is catalyzed by both non-heme and heme carotenoid hydroxylases. In Arabidopsis leaves, the complete complement of β-carotene hydroxylases is encoded by three genes: two encoding non-heme iron hydroxylases (*CHY1*, *CHY2*) and one encoding a cytochrome P450 (*LUT5*). Arabidopsis *chy1chy2lut5 *mutants completely lack β-xanthophylls (zeaxanthin, antheraxanthin, violaxanthin and neoxanthin), while double *chy1chy2 *mutants show approx. 70% reduction in the same compounds in leaves [4,5]. The tomato *wf *mutant, which maps to the *CHY2 *gene, is sufficient to severely impair flower β-xanthophyll biosynthesis, while leaf β-xanthophyll levels remain similar to those found in wild-type plants [6]. In keeping with this result, the tomato *CHY2 *transcript is expressed preferentially in flowers, while the *CHY1 *transcript is expressed preferentially in leaves [6]. These results indicate that, in different tissues, the hydroxylation of β-carotene is preferentially performed by different gene products. In order to elucidate the role of *CHY *genes in the hydroxylation of β-carotene in potato tubers, we took a tuber-specific gene silencing approach.

## Results and discussion

In order to verify the tissue-specificity of expression of the genes controlling carotenoid biosynthesis in potato, we conducted Real Time RT-PCR experiments on leaf and tuber RNA. As can be seen (Table 1), the majority of carotenoid gene transcripts are preferentially expressed in leaves, in agreement with the higher carotenoid content of this tissue, while two housekeeping transcripts (*β-TUBULIN *and *UBIQUITIN*) are preferentially expressed in tubers. Notable exceptions to this trend are the *NXS *and *CHY2 *genes, which show higher levels of expression in tubers. The first gene [7] is the ortholog of the tomato *B *gene, encoding a fruit-specific lycopene β-cyclase [8] and of the pepper *CCS *gene, encoding a fruit-specific capsanthin-capsorubin synthase which also possesses lycopene cyclase activity [9]. The second gene is the ortholog of the tomato *Wf *gene, encoding a flower-specific non-heme β-carotene hydroxylase [6]. Thus, in potato the same members of the lycopene β-cyclase and β-carotene hydroxylase gene families, which in other Solanaceae are preferentially expressed in chromoplast-containing tissues, are preferentially expressed in the tuber. This is an indication that carotenogenesis in potato amyloplasts may share some regulatory mechanisms with carotenogenesis in tomato and/or pepper chromoplasts.

Given that both *CHY *genes show strong levels of expression in the tuber, we decided to choose, as a silencing fragment, a region showing high (>80%) sequence identity between the two genes (data not shown). This region was amplified from tuber cDNA using specific oligonucleotides (see Methods), inserted, in antisense orientation, under the control of the tuber-specific patatin *B33 *promoter [3,10] and introduced in potato (cv. *Desirée*) using Agrobacterium-mediated transformation [11]. Transgenic plants were selected on kanamycin, the presence of the transgene was confirmed via PCR (see Methods), and six independent transgenic lines (*AS-h*, two plants per line) were acclimated in the greenhouse for tuberization. As controls, we used the original Desirée line used for transformation and one line transformed with a *PB33:GUS *construct [3]. At the end of the life cycle, tubers were harvested and tuber production was evaluated. None of the transgenic lines showed major alterations in tuber weight or number (data not shown).

The carotenoid composition of tubers was analyzed through HPLC (Table 2). The *GUS *line and two *AS-h *lines (lines 5 and 6) did not show relevant changes in carotenoid content, with respect to wild-type *Desirée*. Four *AS-h *lines (lines 1 to 4) showed significant (p ≤ 0.001) increases in total tuber carotenoids, as well as changes in carotenoid composition. In these lines, consistent with the hypothesized silencing of *CHY *genes, the levels of β-carotene showed significant increases (up to 38-fold). Levels of downstream β-β-xanthophylls showed more variable trends: the immediate product of β-hydroxylation, zeaxanthin, decreased 2- to 8-fold, while violaxanthin increased significantly in two out of four lines and neoxanthin increased significantly in one line. The final product of the competing ε-β-branch, lutein, increased 4- to 7-fold in all four "expressor" lines. The colorless biosynthetic intermediate, phytofluene, increased in two of the lines, while the other early intermediates (phytoene, ζ-carotene) were below detection in all lines. Consistently with the tuber-specific nature of the promoter used for the silencing construct, no significant variations in carotenoid or chlorophyll content, with respect to the *Wt*, were observed in leaves of *GUS *or *AS-h *lines (data not shown).

The *AS-h *silencing transcript was detected at high levels (0.4- to 27-fold tubulin) in the tubers of the four *AS-h *lines showing significant changes in carotenoid content (Table 3). The highest levels of expression are observed in line *AS-h 3*, which has the highest total carotenoid levels. In tubers of the two *AS-h *lines with unchanged carotenoid content ("non-expressor" lines), as well as in leaves of all lines, the silencing transcript was below the levels of detection of the Real Time RT-PCR assay. Variability in the expression of introduced genes among independent transformants is a common phenomenon in plant transformation [12] and has been found, at comparable frequencies, in the case of the *B33:AS-e *and *B33:GUS *constructs [3].

**Figure 1:**
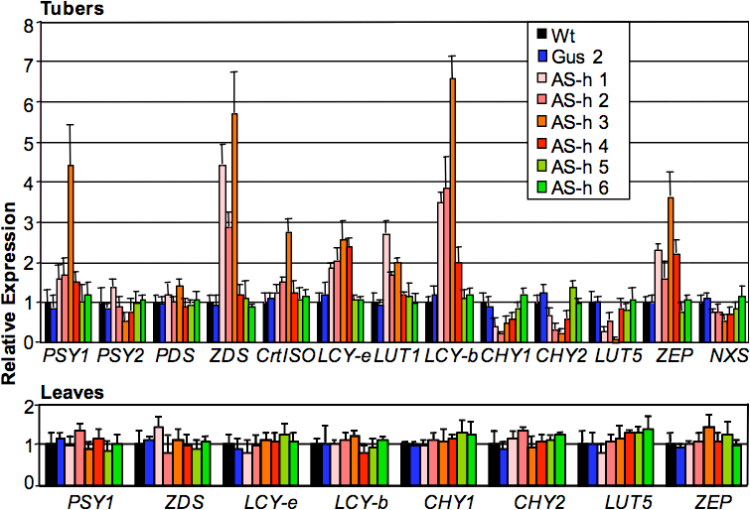
**Endogenous carotenoid gene expression**. Transcript levels were measured through Real Time RT-PCR and were first normalized for expression of the housekeeping β-tubulin gene, and then for the expression levels in the *Wt*. Data show the average and SE (error bars) of determinations from at least 4 different tubers (or leaves) from 2 different plants. For details see Methods.

**Figure 2:**
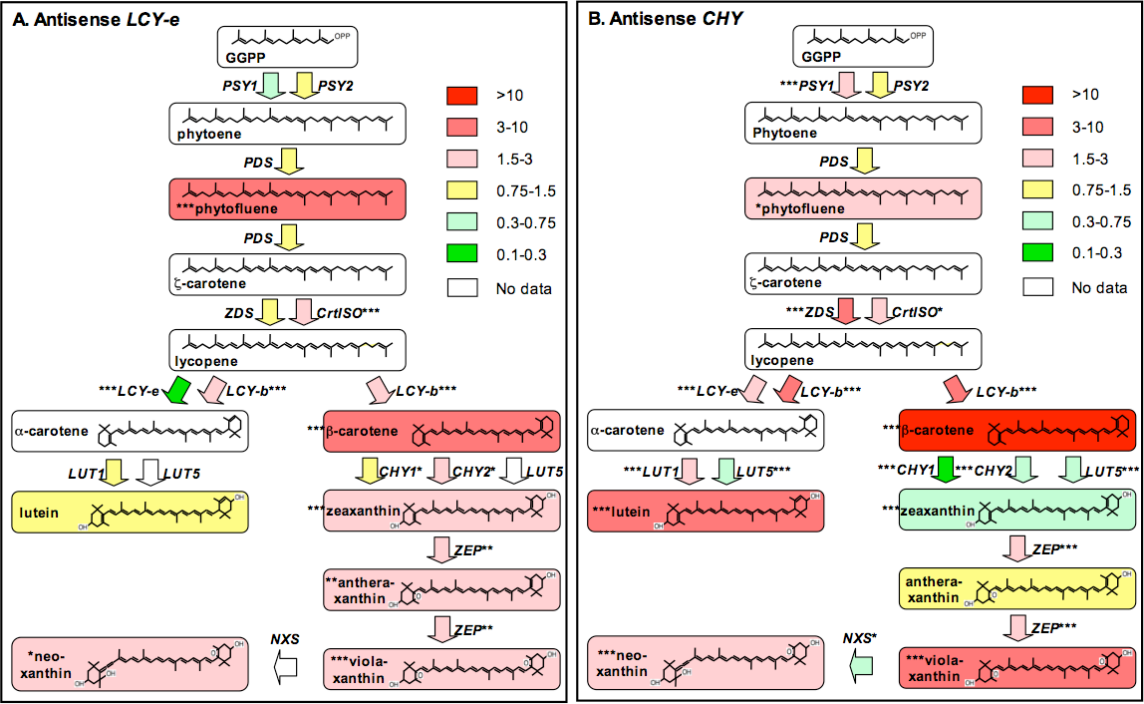
**Schematic representation of metabolite and gene expression changes in engineered tubers**. Boxes represent the metabolic intermediates, arrows represent the genes catalyzing the various reactions. Fold induction or repression with respect to the wild-type – averaged over three transgenic lines- is represented by different hues of red or green, respectively (see legend). White means that no data are available. Asterisks indicate significance of the fold variation with respect to the Wt in an ANOVA test (*: p ≤ 0.05; **: p ≤ 0.01; ***: p ≤ 0.001). (A) *AS-e *lines 1,2,3 [3]. (B) *AS-h *lines 1,2,3 (this paper).

Expression of endogenous carotenoid genes is often altered as a consequence of manipulations modifying the levels of biosynthetic intermediates in the pathway. This phenomenon has been observed both in tomato leaves [13,14] and in potato tubers [2,3]. We measured the expression of carotenoid gene transcripts (*PSY1*, *PSY2*, *PDS*, *ZDS, CrtISO*, *LCY-b, LCY-e, CHY1, CHY2, LUT1*, *LUT5*, *ZEP, NXS*) in tubers, using Real Time RT-PCR. The tuber transcript levels, normalized first for the β-tubulin transcript and then for the *Wt *transcript levels, are shown in Figure 1. The biosynthetic steps catalyzed by these genes are shown in Figure 2. The *GUS *line, as well as "non expressor" lines *AS-h 5 *and *6*, showed only minor variations in gene expression with respect to the *Wt *line. This indicates that culture conditions, somaclonal effects due to regeneration procedures, or the presence of the silencing transgene by itself, do not cause any major variability in endogenous carotenoid gene expression. The endogenous *CHY2 *gene is silenced in the four lines showing alterations in carotenoid content (*AS-h 1 to 4*). Line *AS-h 3*, which is the one showing the highest increase in total carotenoids (Table 2), also shows the most efficient silencing of endogenous *CHY2*. Alongside *CHY2*, also the *CHY1 *transcript is silenced, albeit to different extent, in transgenic tubers of lines *AS-h1 *to *4*. This result indicates that the homology between the two transcripts in the region chosen for silencing is sufficiently high to warrant cross-silencing.

The silencing of *CHY *transcripts causes, directly or indirectly, an extensive remodeling of the expression of the endogenous carotenoid genes. Alongside *CHY1 *and *CHY2*, also *LUT5 *and, to a lesser extent, *NXS *are repressed in lines *AS-h1 *to *4*. The repression of *LUT5 *is likely to have a cooperative effect with the silencing of *CHY1 *and *CHY2 *in mediating β-carotene accumulation, since this gene, like *CHY1 *and *CHY2*, encodes a β-ring hydroxylase [5] (Figure 2). Also, the induction of lycopene beta-cyclase (*LCY-b*) is likely to be enhancing β-carotene content. *LUT5 *and *LCY-b *are, respectively, maximally repressed and induced in line *AS-h3*, which has the highest total carotenoid content. *ZDS*, *LCY-e*, *LCY-b*, *LUT1 *and *ZEP *are induced in lines showing changes in carotenoid content (*AS-h 1 to 4*). *CrtISO *is induced only in line *AS-h 3*, which is one showing the highest total carotenoid levels.

We conducted a comparative assessment of gene expression and of metabolite composition in the carotenoid pathway in the tubers of *AS-e *lines [3] and of *AS-h *lines (this paper). The results are shown in Figure 2 and can be summarized as follows:

• The two lycopene cyclases, *LCY-b *and *LCY-e*, are induced as a result of either manipulation. A notable exception are of course *AS-e *plants, in which the *LCY-e *transcript is silenced as a result of the introduced transgene. We cannot distinguish, at the present moment, whether this induction in *LCY-b *and *LCY-e *transcripts is a consequence of the increase in total carotenoid levels or of the increase of a specific intermediate. Pharmacological experiments with inhibitors of various steps in carotenoid biosynthesis [13,14] could, to a certain extent, discriminate between the different possibilities.

• Another gene showing induction in *AS-e *and *AS-h *tubers is *ZEP*. Zeaxanthin is a rare carotenoid in cultivated potato [15], and the fact that the immediately downstream gene is induced as a result of perturbations in carotenoid content may partially explain this fact. This gene has been silenced in a tuber-specific fashion, resulting in accumulation of zeaxanthin [1].

• A third gene showing induction in both cases is *CrtISO*. Its gene product is involved in the isomerization of *cis *double bonds during the synthesis of lycopene [16-18].

• The overall pool of xanthophylls derived from α- and β-carotene increases in both cases; in *AS-e *lines, lutein levels remain relatively stable, while those of β-β-xanthophylls show a moderate increase; in *AS-h *lines, the product of hydroxylation, zeaxanthin, shows a moderate decrease, antheraxanthin levels are unaltered, while all other compounds show moderate (neoxanthin) to strong (violaxanthin and lutein) increases.

• Early compounds in the pathway are below detection in the *Wt *and in transgenic lines, with the exception of phytofluene, which shows from moderate to strong increases in both *AS-e *and *AS-h *lines.

• By far, the highest increase obtained in both cases is that of β-carotene, one of the main goals of our engineering effort; these results, together with those of Ducreux et al. [2] and Lu et al. [19], clearly show that β-carotene, although it is a very rare compound in cultivated potato [15], can accumulate to remarkable levels after metabolic engineering.

What is the relative contribution of different carotenoid hydroxylases to beta-carotene hydroxylation in tubers? Table 1 shows that the transcripts for all three genes controlling β-carotene hydroxylation in Arabidopsis [5] are expressed albeit at different levels in potato tubers, in the order *CHY2*>*LUT5*>*CHY1*. Comparison of carotenoid content (Table 2) with gene expression (Figure 1) in the different lines suggests that maximal accumulation of total carotenoids, and repression of zeaxanthin content, is observed in line *AS-h 3*, in which *CHY2 *and *LUT5 *are maximally repressed. Thus, both gene expression data in wild-type tubers, and correlations between gene silencing and metabolite levels in transgenic ones, suggest that *CHY2 *is the most important contributor to tuber β-carotene hydroxylation. However, this remains a hypothesis, until accurate measurements are obtained of the levels and activities of the corresponding enzymes.

Recently, Lu et al. [19] showed that the cauliflower Or gene, encoding a DnaJ-related protein, is able to dramatically increase total carotenoid and β-carotene levels in transgenic potato tubers. This approach is complementary to more "classical" ones, like the one reported here, that rely on the alteration of expression, in tubers, of structural genes in the carotenoid pathway, [1][2][3][20][21]. A combination of different approaches holds good promise for the further increase of the provitamin A levels of potato.

## Conclusion

Using an antisense construct under the control of the tuber-specific patatin promoter, we obtained the simultaneous, tuber-specific silencing of the potato *CHY1 *and *CHY2 *transcripts. β-carotene increased and zeaxanthin decreased accordingly in a tuber-specific fashion, while phytofluene, violaxanthin, neoxanthin, lutein and total carotenoids also showed increases. This modification in carotenoid content is paralleled by modifications in endogenous carotenoid gene expression. Modeling of carotenoid gene expression and intermediate metabolite levels in the two cases showed several overlaps with the changes already observed in *LCY-e*-silenced tubers [3]. *CrtISO*, *LCY-b *and *ZEP *were induced in both cases, suggesting that the levels of these transcripts may be sensing the similar changes in metabolite abundance induced by the two types of manipulations (Figure 2). By far the metabolite showing the highest increase in both cases was β-carotene, confirming that this compound is relatively stable in potato tubers.

## Methods

Unless otherwise indicated, molecular biology methods are as described [22]. A 0,56 Kb *CHY2 *cDNA fragment was amplified from potato (*cv Desirée*) tuber cDNA using the primers As-chy Up1 and As-chy Dw2 (Table 4). These primers inserted, respectively, Sac I and Bam HI sites upstream and downstream of the cDNA fragment. After intermediate cloning in the *pBSK+ *vector and re-sequencing, the fragment was inserted, in antisense orientation, to replace the *GUS *gene in the *pBI33:GUS *vector [3].

Potato (*cv Desirée*) was transformed as previously described [11]. Plantlets growing on kanamycin were tested by PCR, using primers AS-h Up and Nos-test 2 (Table 4). PCR-positive, rooted plantlets were adapted in greenhouse in pots (diameter: 25 cm) in a soil mixture composed of 1/3 sand and 2/3 of sterile soil (Terraplant 2, BASF). Photoperiod was set at 14 hours of light and 10 hours of dark, with temperature set at 24°C during the light period and at 16°C during the dark period. In the advanced phase of growth, the day temperature was kept around 20°C in order to promote tuberization. During tuberization, irrigation was reduced in order to prevent tuber decay.

Tubers from the lower 2/3 of the pot ("deep" tubers) were collected separately from superficial ones, washed in water, briefly dried at room temp, cut in pieces and frozen at -80°C. Tuber productivity for each line was estimated as the total number of tubers produced and as their total weight. All carotenoid and RT-PCR measurements were conducted on at least 4 different "deep" tubers per each line, to prevent possible alterations in carotenoid composition/gene expression resulting from light accidentally illuminating the superficial tubers.

Total RNA was isolated from frozen tissue and analyzed through Real Time RT-PCR using previously published methods [13,23]. Two independent RNA extractions and four cDNAs (two from each RNA) were used for the analyses; RT-PCR conditions and gene-specific primers were as in Diretto et al (2006) with the addition of the following genes: Lut5 (ESTID18235) and NXS (AJ272136). Real Time assay oligos for these genes are described in Table 4. In order to discriminate the introduced *AS-h *mRNA from the endogenous *CHY *mRNA, the former was amplified using primers *AS-h *RT Up and *AS-h *RT Dw, while the latter was amplified using primers Chy1 Up and Dw, and Chy2 Up and Dw (Table 4).

HPLC analysis was performed exactly as described previously [3].

Statistical analysis (one-way ANOVA) was performed using the PAST software [24].

## Authors' contributions

GG, RT and PB planned and supervised the work. FM prepared the constructs for transformation, RT performed the transformations and maintained the lines in vitro, DP and GD grew and sampled the plants, GD performed the Real Time RT-PCR assays and the statistical analysis, RW performed the HPLC's. All authors read and approved the final manuscript.

## Figures and Tables

**Table 1:** Tissue-specific expression of carotenoid biosynthesis genes in potato

	**Leaves**	**Tubers**
*Tubulin*	5.07 ± 2.17	46.18 ± 19.54
*Ubiquitin*	271.42 ± 83.97	417.30 ± 108.97
*Psy1*	161.33 ± 27.44	55.85 ± 22.64
*Psy2*	33.82 ± 5.31	10.28 ± 1.62
*Pds*	46.9 ± 16.41	3.71 ± 0.13
*Zds*	3.18 ± 1.20	0.19 ± 0.04
*CrtISO*	29.04 ± 12.09	7.07 ± 1.54
*Lcy-e*	1115.65 ± 482.19	3.15 ± 0.89
*Lcy-b*	20.52 ± 6.38	4.73 ± 0.93
*Lut1*	7.93 ± 2.32	4.73 ± 1.36
*Chy1*	70.65 ± 27.59	14.13 ± 1.06
*Chy2*	49.39 ± 21.24	61.48 ± 19.07
*Lut5*	121.34 ± 32.15	25.26 ± 12.89
*Zep*	841.85 ± 114.78	15.93 ± 1.22
*Nxs*	0.85 ± 0.05	14.24 ± 6.59

Transcript levels were studied via Real Time RT-PCR, using gene-specific oligonucleotides on RNAs isolated from a minimum of 4 different tubers or leaves from 2 different wild-type plants. Numbers indicate attograms gene-specific cDNA/20 ng total RNA. For details, see Methods.

**Table 2:** HPLC analysis of tuber carotenoids (ng/g dry weight)

	**Phytofluene**	**Lutein**	**β-carotene**	**Zeaxanthin**	**Antheraxanthin**	**Violaxanthin**	**Neoxanthin**	**Other Xanth.**	**Esters**	**Total**
**Wild-type**	61.69 ± 5.60	426.00 ± 124.44	2.25 ± 1.17	320.87 ± 111.63	1415.67 ± 335.02	1598.73 ± 468.72	468.76 ± 161.43	76.91 ± 22.60	517.06 ± 178.15	4887.95 ± 1421.16
**Gus 2**	54.30 ± 5.28	501.81 ± 126.33	3.11 ± 2.15	311.42 ± 98.85	1317.06 ± 149.48	1282.35 ± 403.34	488.24 ± 121.55	91.14 ± 21,55	615.01 ± 98.73	4664.44 ± 1022.00
**Fold Variation**	**0.88**	**1.18**	**1.38**	**0.97**	**0.93**	**0.80**	**1.04**	**1.18**	**1.19**	**0.95**
**As-h1**	162.57 ± 2.88	1590.36 ± 257.09	85.30 ± 2.33	168.37 ± 308.48	2198.74 ± 983.80	3452.99 ± 749.84	1509.40 ± 269.05	177.44 ± 54.47	4918.76 ± 920.25	14263.95 ± 2992.78
**Fold Variation**	2.63***	**3.73**	37.91***	0.52*	**1.55**	2.16*	3.22***	2.31*	9.51***	2.92***
**As-h2**	55.64 ± 0.58	2637.17 ± 570.03	34.08 ± 11.71	147.90 ± 39.91	945.83 ± 156.85	802.98 ± 87.20	832.59 ± 240.51	367.85 ± 19.18	4587.20 ± 1069.79	10411.26 ± 2195.20
**Fold Variation**	**0.90**	6.19***	15.15**	0.46*	**0.67**	**0.50**	**1.78**	4.78***	8.87***	2.13**
**As-h3**	74.70 ± 8.85	2982.04 ± 881.18	56.34 ± 16.90	37.09 ± 5.84	1034.68 ± 402.16	11422.26 ± 2470.47	754.32 ± 228.82	329.06 ± 87.63	5068.07 ± 1181.94	21758.57 ± 5274.94
**Fold Variation**	1.21*	7.00***	25.04***	0.12***	**0.73**	7.14***	**1.61**	4.28***	9.80***	4.45***
**As-h4**	67.64 ± 24.22	1808.90 ± 239.24	32.65 ± 6.50	153.71 ± 39.99	1102.94 ± 287.98	3377.31 ± 371.15	764.34 ± 171.39	255.47 ± 17.75	1789.33 ± 51.88	9352.31 ± 1186.71
**Fold Variation**	**1.10**	4.25***	14.51**	0.48**	**0.78**	**2.11**	**1.63**	3.32***	**3.46**	1.91**
**As-h5**	63.30 ± 5.84	764.54 ± 244.69	2.71 ± 7.75	218.53 ± 140.68	1382.88 ± 203.52	2018.71 ± 282.39	456.06 ± 55.76	126.36 ± 3.23	1293.42 ± 564.92	6326.51 ± 1502.94
**Fold Variation**	**1.03**	**1.79**	**1.20**	**0.68**	**0.98**	**1.26**	**0.97**	**1.64**	**2.50**	**1.29**
**As-h6**	73.12 ± 19.18	575.11 ± 71.52	4.31 ± 0.43	331.03 ± 28.14	1551.15 ± 108.39	1780.21 ± 509.03	617.62 ± 103.11	120.02 ± 35.45	924.89 ± 132.96	5977.47 ± 989.06
**Fold Variation**	**1.18**	**1.35**	**1.92**	**1.03**	**1.10**	**1.11**	**1.32**	**1.56**	**1.79**	**1.22**

Carotenoids were measured via diode array HPLC (see Methods) on a minimum of 4 different tubers from 2 different plants. Fold variation with respect to the wild-type is reported for each carotenoid compound and for each line. Asterisks indicate significance of the fold variation in an ANOVA test (*: p ≤ 0.05;**: p ≤ 0.01; ***: p ≤ 0.001). For details, see Methods.

**Table 3:** Trangene expression.

**Line**	**Organ**	***AS-chy *expression**
		
		**(Fold Tubulin)**
*Wild-type*	Leaf	nd
	Tuber	nd
*Gus 2*	Leaf	nd
	Tuber	nd
*AS-h1*	Leaf	nd
	Tuber	7.428 ± 1.228
*AS-h2*	Leaf	nd
	Tuber	10.721 ± 3.152
*AS-h3*	Leaf	nd
	Tuber	27.630 ± 8.457
*AS-h4*	Leaf	nd
	Tuber	0.412 ± 0.046
*AS-h5*	Leaf	nd
	Tuber	nd
*AS-h6*	Leaf	nd
	Tuber	nd

*AS-h *transgene expression was measured via Real Time RT-PCR and normalized for *the β-TUBULIN *transcript. For details see Methods. nd = not detectable.

**Table 4:** Primers used

**Primer name**	**Sequence**	**Use**
AS-chy Up1	TAGAGCTCGGGATTACTTC	AS-chy cloning
AS-chy Dw2	ATGGATCCTCCTTTTCCAA	AS-chy cloning
AS-h Up	GTTAAGGGAACTTCTCCAC	PCR screening
Nos-test 2	CGCGTATTAAATGTATAATTG	PCR screening
AS-h RT Up	ACCCTCCATTTGCCACGAA	Real-time assay
AS-h RT Dw	TTATATGATAATCATCGCAAGACCG	Real-time assay
Chy1 Up	CTTGGCCCAAAACCCACTT	Real-time assay
Chy1 Dw	CCTCAAATTGAGGTTTCAGCTTCT	Real-time assay
Chy2 Up	TTTTGCTGTCTCGAAGAAAGCC	Real-time assay
Chy2 Dw	AGCCAACAGGCAGCTAAACTCT	Real-time assay
Lut5 Up	GTCTCAAGCAAGCAACTTCGTG	Real-time assay
Lut5 Dw	GATAAAAGGTCCATGTGAGCACTG	Real-time assay
Nxs Up	CTTGGAGGAGACTTCTTTGGTGA	Real-time assay
Nxs Dw	CGGAAGTGGTCCTCCCATAG	Real-time assay

Sequences of the primers used for cloning of the gene fragment, for PCR screening of the putative transgenic plants, and for Real Time RT-PCR quantitation of transcript levels. For further details, see Methods.
